# A model for the cognitive assessment of physicians

**DOI:** 10.3389/fpubh.2025.1555950

**Published:** 2025-07-16

**Authors:** Victor A. Del Bene, George Howard, David S. Geldmacher, Elizabeth Turnipseed, Catherine Brown, Kathleen Lowry, Trevor Starling, Kate Bryan, T. Charles Fry, Keith A. Jones, Ronald M. Lazar

**Affiliations:** ^1^Department of Neurology, Heersink School of Medicine, The University of Alabama at Birmingham, Birmingham, AL, United States; ^2^The Evelyn F. McKnight Brain Institute, Heersink School of Medicine, The University of Alabama at Birmingham, Birmingham, AL, United States; ^3^Department of Biostatistics, School of Public Health, The University of Alabama at Birmingham, Birmingham, AL, United States; ^4^Department of Medicine, Heersink School of Medicine, The University of Alabama at Birmingham, Birmingham, AL, United States; ^5^The University of Alabama Health Services Foundation, P.C, Birmingham, AL, United States; ^6^Department of Anesthesiology, Heersink School of Medicine, The University of Alabama at Birmingham, Birmingham, AL, United States

**Keywords:** impaired physicians, aging physicians, cognition, neuropsychology, competence

## Abstract

**Introduction:**

With aging in the larger population and physician workforce, there has been growing emphasis on physician cognitive impairment. We propose that the determination of cognitive status in physicians, regardless of the cause, should be based on a comparison to other physicians, rather than against the non-physician populations. Our objective was to develop a normative database of healthy physicians that can be used in physician competency evaluations.

**Methods:**

This study was a prospective cross-sectional observation design. Cognitive test data from 190 healthy physicians between the ages of 35 and 65 without work-related concerns was collected in an academic medical center neuropsychology clinic. Our primary outcome was performance on a neuropsychological test battery. All performances were z-score transformed (*Mean* = 0, *SD* = 1).

**Results:**

When comparing the distribution of our physician sample to the average performance of non-physician 45-year-olds, the average physician performance is skewed to the right, indicating group-level physician performances of 0.5 to 1.0 standard deviations higher than the general population. For overall cognitive performance, multivariate regression revealed older age (−0.18, 95%CI −0.24 −0.13, *p* < 0.0001) was associated with lower overall cognitive performance, but still better than the average 45-year-old, non-physician group.

**Discussion:**

In conclusion, physicians outperformed the general public on tests of cognitive functioning. Even older physicians (ages 60–65) performed above the average general population 45-year-old, reflecting preserved cognitive abilities. Existing age-corrected methods from the general population can potentially mask cognitive impairment in medical professionals.

## Introduction

The population of the US is older than it has ever been, including those still working. It was recently reported that the growth among those 65 + years and older who continue to work is driven both by their growing prevalence and the proportion still having jobs (1). Physicians are well represented in this older cohort, with one estimate that within the decade, more the 2 of every 5 physicians will be older than 65 years (2). With the aging of physicians, there is increasing concern about cognitive impairment, whether from illness, injury or just aging that threatens the capacity to practice medicine (3–11). Whereas these changes may be more common among older physicians, the broader question arises about cognitive competence (i.e., cognitive abilities) of physicians at any age.

The matter of physician competency arises commonly from a complaint by a patient, colleague, trainee, or other healthcare provider based on medical error or the observation of motor or cognitive issues involving memory, thinking speed, or decision-making. When the problems seem to be cognitive in nature, the usual practice is to refer individuals for a neuropsychological (sometimes referred to as a neurocognitive) assessment to evaluate multiple cognitive domains, which has been demonstrated to have the sensitivity to determine the cognitive status of physicians (12–15). When non-physician patients are referred for neuropsychological examination in general clinical practice, normative data are used from a demographically similar population, including age and education, as a basis of comparison to determine the individuals relative cognitive state. Lower scores that represent greater deviations from the standard indicate greater impairment. In this respect, the goal is to insure that a 65-year-old is not being compared to a 25-year-old on skills which are expected to decline with aging (16).

We propose that the application of age correction to address physician cognitive impairment falls short on two accounts: First, we assumed that physicians have higher cognitive skills than the general population (15), and second, we expect that practicing physicians should have the same level of cognitive competency regardless of age, or any other demographic factors. It would therefore seem reasonable that we need to have a peer group of otherwise healthy physicians aged 35 to 65 years old comprise the standards against which to judge empirically the cognitive performance of physicians whose cognition is in question. Given such data, we would validate the assertion that those in the physician normative group at least in our institution are functioning significantly better on a battery of cognitive tests than the general population. Secondarily, we could use this physician normative group for a similar comparison across different cognitive domains.

## Methods

### Participants and recruitment

We recruited healthy, practicing physicians, ages 35 to 65 years old, employed at the University of Alabama at Birmingham (UAB) Heersink School of Medicine, without any health and occupational concerns. This study was approved by the UAB Institutional Review Board. Enrollment began in January 2022 and ended October 2023. A detailed description of our study goals and methodology has been previously reported (17).

To minimize sampling bias, we received a list of all physician names and email addresses employed at the medical center. We divided the list into four subgroups based on the listed medical specialty: (1) general medicine and psychiatry, (2) board specialists in medicine, (3), intensivists and interventionists, and (4) surgeons. The names within each group were then randomized and a recruitment email was sent to physicians in sequential order.

All participants provided demographic and professional information including age, gender, and medical specialty. We also included a question pertaining to whether they were on call or just finished being on call at the time of testing. Out of concern that physicians might not want to participate in a cognitive study at the institution where they are employed, we felt it was critical to assure privacy to all study participants, ensuring there was no way their employer could access their data. Therefore, race and ethnicity were not collected to maintain confidentiality. Moreover, all data were immediately de-identified, entered in a RedCap database, and the physical cognitive protocols were destroyed. It was also conceivable that a physician might volunteer because of concern of declining cognition. To address this possibility, a cognitive screener (Montreal Cognitive Assessment; MoCA) (18) was administered at the end of the cognitive test battery (see below). If physicians performed in the impaired range on the MoCA (< 20), the study protocol was to inform them immediately and instruct them to contact their primary care provider for further medical evaluation. These tests batteries would then be discarded.

### Measurement of cognition

All participants underwent an in-person, neuropsychological test battery administered by an experienced psychometrician and comprised of 11 standardized, validated measures of cognitive function (19). The details of this battery have been described elsewhere (17) and the cognitive test battery is very similar to prior work from our group (15). In brief, the cognitive domains were processing speed, attention/working memory, language, visuospatial skills, verbal memory, visual memory, executive function, and motor processing speed/fine motor dexterity. Depressive symptoms can have an effect on cognition, which was evaluated with the eight-item Patient Health Questionnaire (20). To test the hypothesis that the physician group would perform significantly better than the general population, we chose the mean and standard deviations of the published standards for 45-year-old non-physicians, based on the average age of our physician cohort was approximately 46 years old (See Table 1). The physician performances on each test were z-scored transformed using the norms of the 45-year-old comparison group, with higher z-scores representing better scores. When there were two tests for a given domain, an average was computed from the z-scores for that domain. Our primary outcome was the neuropsychological test battery composite z-score for the physician group derived from the average z-score from all domains. The score from the MoCA was not included in the composite score.

**Table 1:** Demographic factors.

Variables	Physician Groups		
	All	Group 1	Group 2
*n*	190	115	75
Age	46.1 (9.0)	46.6 (9.2)	45.4 (8.8)
Sex (% male)	61.1%	53.9%	72.0%
Handedness (%)			
Right	85.8%	91.3%	77.3%
Left	5.3%	5.2%	5.3%
Ambidextrous	8.9%	3.5%	17.3%
MoCA	27.9 (1.8)	28.2 (1.7)	27.5 (2.0)
PHQ – 9 (Patient Health Questionnaire-9)	1.9 (2.4)	1.9 (2.3)	1.9 (2.5)

Group 1 = Generalists, psychiatrists, and board-certified specialists in medicine; Group 2 = Intensivists, interventionists, and surgeons. Numbers in parentheses are standard deviation values.

### Statistical analysis

As previously described (17), each of the individual cognitive assessment was standardized to the general population performance at age 45. Multiple linear regression was used to analyze the relationship of demographic and professional characteristics with cognitive performance. Alpha was set at 0.05. For each cognitive domain, we plotted the distribution of our physician sample relative to the mean performance of a 45-year-old general population norms.

## Results

In all, 190 physicians were enrolled. Among our cohort, there were 30 in general medical practice, psychiatry, and physiatry, 85 in boarded non-surgical medical specialties, 33 interventionist and intensivists, and 42 surgeons. While our goal was to have four equal groups, (A) general medicine and psychiatry, (B) board specialists in medicine, (C) intensivists and interventionists, and (D) surgeons, ultimately, the UAB institutional distribution of physician specialties did not support our original enrollment assumptions. Because the pool of proceduralists were fewer, before we analyzed the data, we felt it reasonable to merge groups A and B to form Group 1 (general medicine, psychiatry, and board specialists in medicine), and groups C and D became Group 2 (intensivists, interventionists, and surgeons). In cases where there was potential overlap for Groups 1 and 2, we asked the physician if their role required procedural skills. If they responded with yes, then they were included in Group 2. See Table 2 for the distribution across medical specialties.

**Table 2:** Medical specialties constituting physician groups.

Planned physician grouping	Medical specialties
General Medical Practice & Psychiatry	Internal Medicine, Family Medicine, Preventive Medicine, Primary Care, General Pediatrics, Adolescent Medicine, Psychiatry
Board Specialists in Medicine	Cardiology, Clinical Immunology & Rheumatology, Dermatology, Endocrinology, Gastroenterology & Hepatology, Geriatrics/Gerontology & Palliative Care, Hematology & Oncology, Infectious Disease, Neurology, Obstetrics & Gynecology (non-surgical), Ophthalmology (if only outpatient), Pathology, Pulmonology, Physical Medicine & Rehabilitation Services, Radiology (Diagnostic)
Intensivists & Interventionists	Anesthesiology & Perioperative Medicine, Emergency Medicine, Critical Care, Hospital Medicine, Ophthalmology (if performs procedures), Otolaryngology Services, Radiation Oncology, Radiology (Interventional), Renal Transplant Services, Urology
Surgeons	Neurosurgery, Obstetrics & Gynecology (surgical), Oral & Maxillofacial Surgery, Orthopedic Surgery, Surgery Service (general, cardiac, transplant, plastic surgery, etc).

These were the four groups we planned to stratify the sample by prior to studying the enrollment distributions. Ultimately, we condensed this to Group 1 (Group A & B) and Group 2 (Group C & D), based on the distinction of the physician being a proceduralist or non-proceduralist.

### Overall cognitive performance

**Figure 1:**
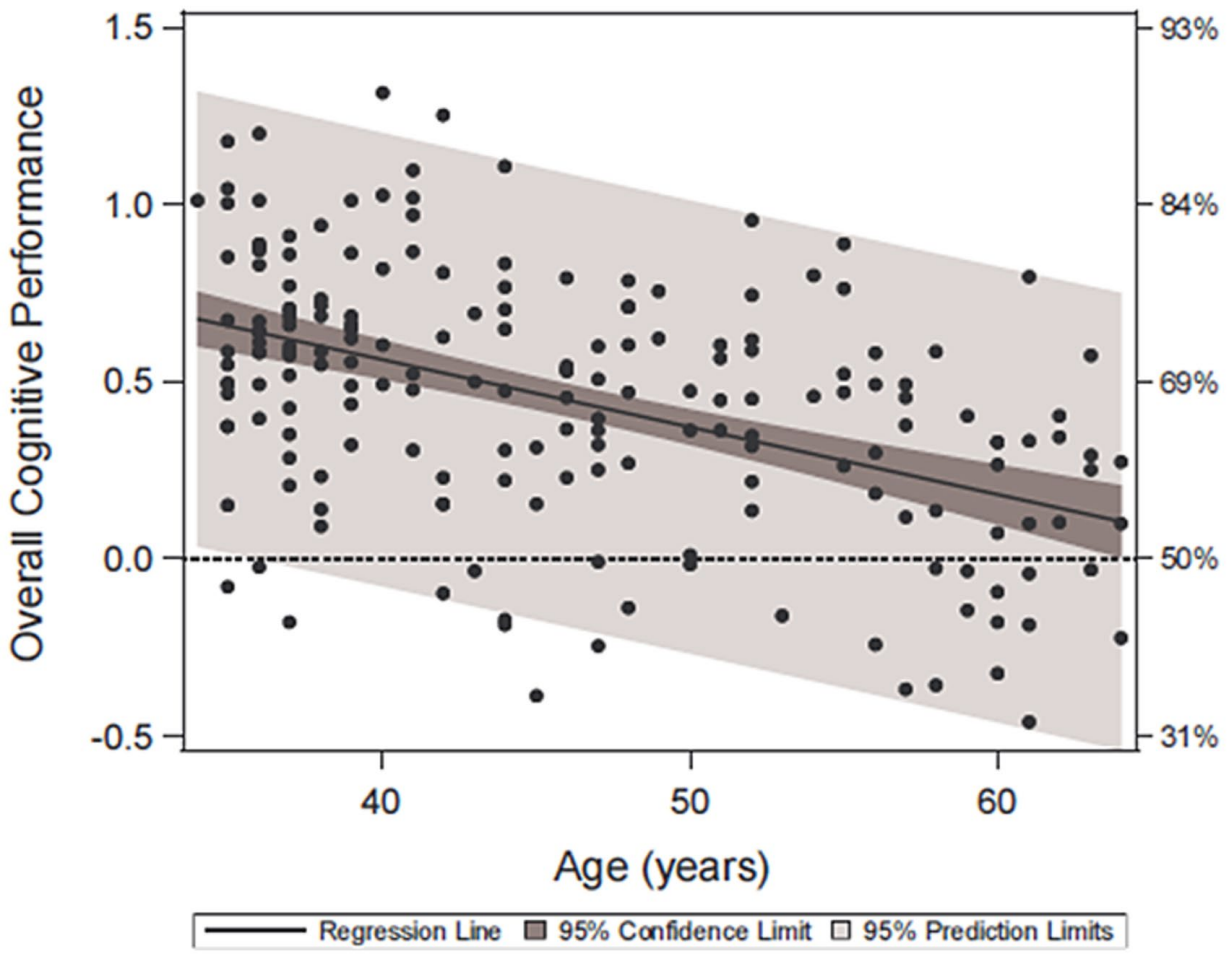
Overall cognitive performance across age. Scatter plot and regression relationship between overall physician cognitive performance (standardized or “z-scored” to the general population at age 45) with the physician age. The left vertical axis is the cognitive performance z-score, and the right vertical axis is the percent of the general population at that score or less (for example, 84% of the population will fall below a z-score of 1.0). The 95% prediction limits show the range of performance within which 95% of the physicians should perform as a function of age.

Figure 1 depicts the cognitive performances of the physician by age relative to the 45- year-old, non-physicians for the composite score of the cognitive domains. Multivariate regression revealed only 10-year older age (−0.18, 95%CI −0.24 −0.13, *p* < 0.0001) was associated with lower overall cognitive performance. Importantly, 86% (163/190) scored above the 50^th^ percentile for the 45-year-old comparison group. Even at older ages, no physician scored at a level that would be regarded as impaired (≤5th percentile) based on conventional standards for the non-physician population. The 95% confidence limits show the bounds for the regression line, which falls above the z-score of 0.0 for all ages, indicating that regardless of the physician age, the average performance of the physicians was significantly (*p* < 0.05) above the average performance of the general population at age 45. There were no significant associations with medical specialty (*p* = 0.57), gender (*p* = 0.72), or being on call (*p* = 0.47).

### Cognitive domains

**Figure 2:**
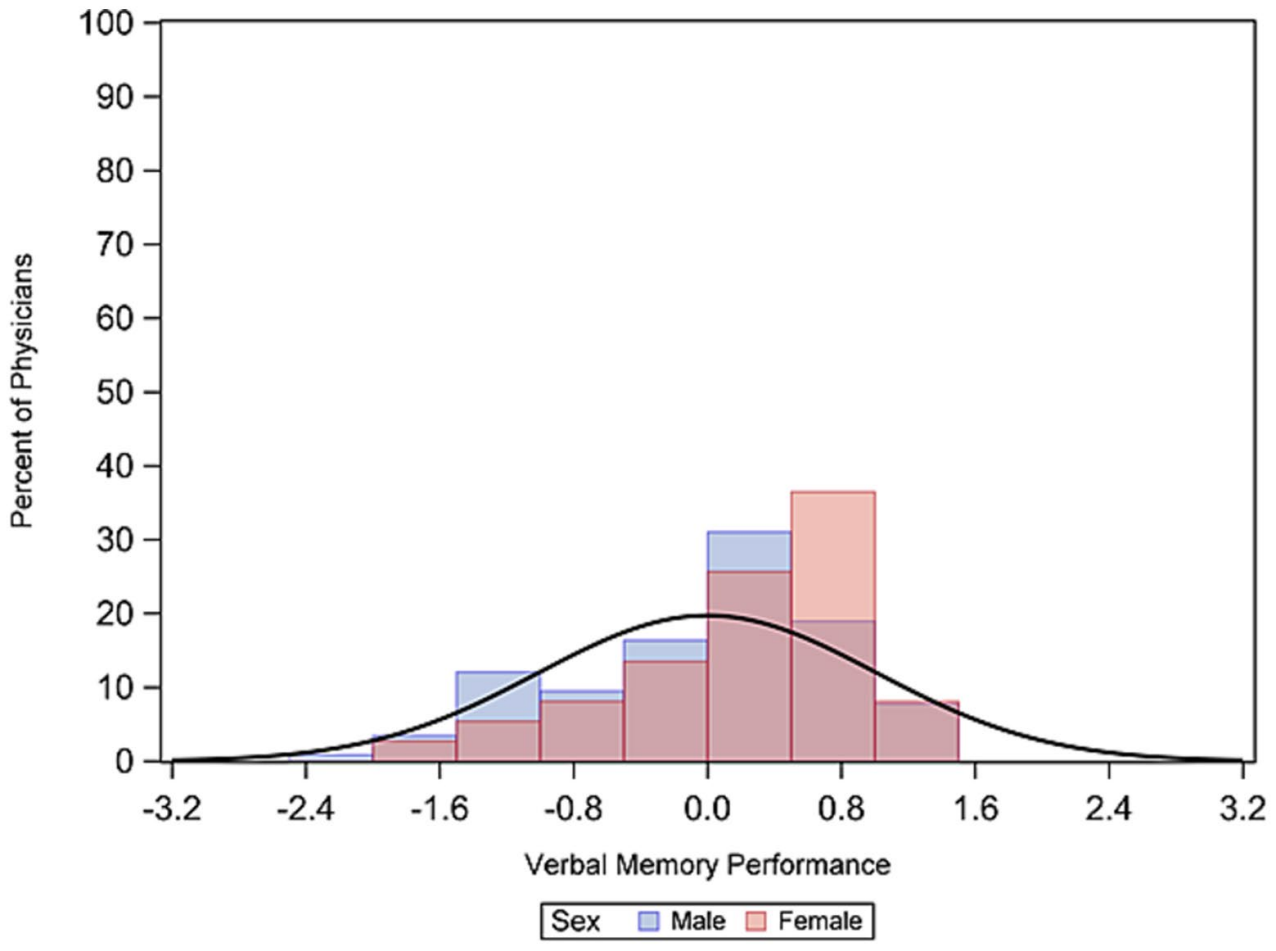
Verbal memory gender differences. Percent of male and female physicians scoring at different z-score bins.

**Figure 3:**
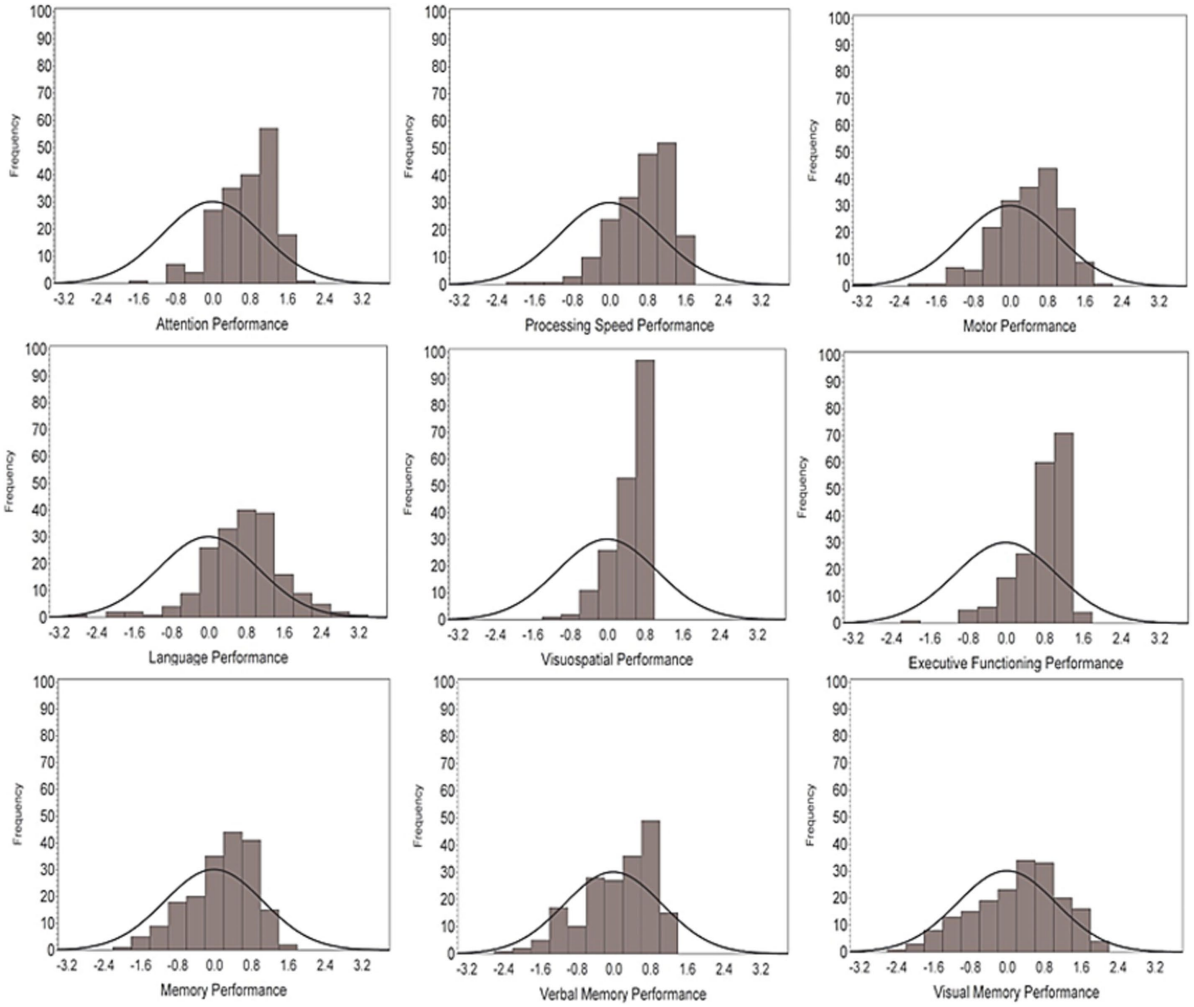
Distributions of performances by cognitive domains. A z-score of zero reflects the average performance of a non-physician 45-year-old, with the bell curve representing the theoretical distribution of the general public. The frequency histogram bars representing the distribution of scores in our physician cohort.

Age-related changes were observed across most cognitive domains. Multivariate regression models revealed, that a 10-year older age was associated with slower motor speed and dexterity (−0.32, 95%CI −0.43 −0.21 *p* < 0.0001), slower processing speed (−0.25 95%CI −0.34 −0.16 *p* < 0.0001), reduced attention and working memory (−0.27 95%CI −0.36 −0.19 *p* < 0.0001), reduced memory (−0.21 95%CI −0.33 −0.10 *p* = 0.0003), and lower performance on tests of executive functioning (−0.20 95%CI −0.28 −0.12 *p* < 0.0001). Older age was not associated with verbal memory (−0.12 95%CI −0.24 0.01 *p* = 0.068) or visuospatial performance (−0.03 95% CI −0.10 0.03), but female physicians outperformed male physicians for verbal memory performance (0.25 95%CI 0.02 0.49 *p* = 0.034) (Figure 2). This verbal memory gender difference was further explored, with no effect of age (−0.10 95%CI −0.23 0.02, *p* = 0.11), or an age by gender interaction (*p* = 0.76) seen in statistical analyses. Physician group, gender, or being on call were not associated with motor speed, processing speed, attention/working memory, memory, and executive functioning. No factors were associated with performance on tests of language or visuospatial processing. Even though age was associated with lower performance across several cognitive domains, the distribution of scores, as seen in Figure 3, are skewed right, indicating physician group-level performances 0.5 to 1.0 standard deviations higher than the average 45-year-old from the general population. Attention, processing speed, language, visuospatial, and executive functioning performances for physicians were all significantly above the average score for 45-year-olds from the general population (*p* < 0.0001). The physician’s overall memory composite was also greater than the average performance from the general population (*p* = 0.0033), with this difference explained by stronger visual memory in physicians (*p* = 0.0013), while physician verbal memory performance did not significantly differ from the general population at age 45 (*p* = 0.15).

## Discussion

To make empirically based decisions with regard to cognitive competency, it is reasonable to have an occupationally-relevant reference group to reduce the risk of invalid determinations regarding cognitive impairment. In this study, we report neuropsychological normative performances for healthy physicians, ages 35 to 65, from a single academic medical center in the United States, and factors associated with cognitive performance. At a group-level, our physician cohort performed around 0.5 to 1.0 standard deviations above the average non-physician 45-year-old. We also found that older age was modestly associated with lower cognitive performance, but even with this association, our 60- to 65-year-old physicians still performed above healthy 45-year-old non-physicians. Age was not associated with verbal memory performance, but female physicians modestly outperformed their male colleagues. No other measured professional or demographic factors were associated with cognitive performance. This study reflects a significant step forward in improving the validity of physician fitness-for-duty cognitive assessments and serves as a model for collaborative efforts to increase the robustness and generalizability of the normative sample.

In general practice, the purpose of age-corrected normative standards is to avoid attributing decline to pathology when normal aging may be responsible for changes on some tasks. Because the determination of competence is a dichotomous outcome, the underlying mechanism for decline becomes less relevant: either the physician is competent or not, even if the underlying reason for decline may potentially be reversible. If someone is impaired because of an illness, injury, mood symptoms, or even aging (4, 21, 22), at a minimum there should be a temporary pause on clinical practice until further evaluation reveals the underlying cause and possible treatment. While previous work took this approach in a very small sample in a single medical specialty (15), a recent study reported age-corrected normative data for older physicians (ages 60–78) (11). The risk of age correction is that the standards to continue practice are lowered with increasing age, so that competence as a threshold to practice is defined at different levels across ages.

Consistent with prior research that demonstrated higher full-scale IQ scores in healthy physicians (15), here we demonstrated that at a group-level, healthy physicians typically perform around 0.5 to 1.0 standard deviations above non-physicians on cognitive tests. The implication is that the likelihood of detecting true cognitive decline in physicians based on normative standards for the general population is greatly reduced. Such false negative determinations places patients at risk for less-than-optimal care.

Commercial airline pilots are highly regulated by the Federal Aviation Administration, with mandatory retirements of airline pilots after age 65 (23). In pilots, working memory, reasoning, and impulsivity were associated with flight simulator performance (24). Cognitive assessment is a better predictor of flight performance than chronological age (25), but increased age does increase the likelihood that a pilot will have an impaired cognitive profile (26). The public health parallels between pilot and physician cognitive impairment are clear – impairment for any reason can result in the harm of the people they are safeguarding or treating. Surgeons in the United Kingdom, Japan, India, China, and Finland all have mandated retirement ages (22, 27). Based on our findings, we feel that age-mandated retirement of physicians may not be the correct approach for several reasons. First, our 60-65-year-old physician cohort still outperformed people 15 years younger, but we will need longitudinal data before we can determine the typical rate of decline in high-functioning physicians. Second, there is a physician shortage (28) and mandated retirement because of an arbitrary age cutoff will exacerbate this problem, decrease access to care, and remove skilled, knowledgeable providers that need to train the next generation of physicians. Age-mandated assessment approaches have also come under legal scrutiny (29). There are also infrastructure concerns of broad age-mandated assessments in large medical centers, primarily the limited number of clinical neuropsychologists who can complete this work while also addressing the growing clinical demands of cognitive impairment in an aging population (30). Currently, we propose that a fitness-for-duty evaluation following a clinical error, which raises to the level of concern, is a conservative approach. Due to concerns pertaining to ecological validity and how cognitive tests directly relate to real-world clinical practice, the cognitive assessment is only the first step of the evaluation process. Correlating the nature of the alleged work-related errors with the pattern of deficits observed on neuropsychological assessment is always necessary. If a physician performs poorly on the neuropsychological test battery, we are reasonably confident that they should not practice medicine, either indefinitely, or until a reversible cause is treated. If there are work-related errors, but they perform well on the neuropsychological test battery, then the next step might be simulation models and case studies, which are now standard in medical education (31). If a referred physician passes this phase, then real-world observation would seem appropriate.

The observed verbal memory gender difference in our physician cohort is consistent with prior research from non-physician samples. On average, cognitively healthy women outperform their male peers on neuropsychological tests of verbal memory (32). The female advantage appears to be related to stronger semantic clustering, the use of covert rehearsal, and the use of visualization and method of loci strategies (i.e., placing the words in a familiar environment) (33). A recent meta-analysis investigating verbal fluency and verbal memory gender differences found small effect sizes of females having stronger verbal memory than males (range of Cohen’s d values = 0.02 to 0.42; 13 of 16 studies had Cohen’s d values < 0.3). However, as these authors comment, there are a number of biases in the literature, such as published studies showing larger effect sizes than unpublished studies (Cohen’s d values ranging from 0.09 to 0.39) (34). In our study, female physicians outperformed their male colleagues, but with a modest effect consistent with the published literature. Compared to prior studies on this topic from the general public, our sample is unique with higher educational attainment. The same cognitive strategies outlined above (33) may or may not apply to this cohort. While this is likely a true finding based on prior research, the novelty of this cohort suggests the finding will need to be replicated in an independent sample of healthy physicians. Finally, the observed verbal memory gender difference was not due to the female physician participants being younger than their male colleagues.

Limitations of this study include collecting data from a single academic medical center, which may limit the generalizability of our findings. However, our sample only included board-certified physicians licensed to practice medicine, ensuring similar academic and training standards compared to other institutions. While it is possible that physicians outside of academic medicine may perform differently on these neuropsychological tests, it is not likely there will be large differences because of the multiple cognitively demanding milestones needed to become a physician (i.e., college, MCAT, medical school, residency, fellowship). Not all training programs and physician trajectories are identical and surely there will be variability in cognitive abilities amongst physicians, but the educational standards ensure a basic minimum capability has been met and lowers the risk that one group of physicians will significantly outperform another group. It is also possible that the physicians at an academic training institution are not representative of the general physician population. The cognitive demands at an academic medical center may potentially differ from other settings. Future research will be needed to explore this further, but physicians will often choose different settings to work for multiple reasons that are independent of innate abilities such as location and proximity to family, student loan repayment programs, patient population, amongst other factors. Another possibility is that physicians recruited to our study but who ultimately decided not to participate might have had concerns regarding their cognitive function. Rather, if this bias exists, then we are more confident that the data were derived from those who were more likely to be cognitively intact, which was necessary to ensure the sample was free of cognitively impaired physicians. Another potential limitation is the lack of longitudinal data because we did not track participant trajectories over time. Such a model would help us better understand the cognitive aging process in healthy physicians and would allow us the possibility of linking cognitive declines with work-related issues that emerge over time. We included the “on call variable” to capture fatigue, but we do not have quantifiable datapoints on workload, sleep deprivation, or burnout, which be addressed in future research as these can all affect cognition. Finally, the lack of diversity and demographic data (race, ethnicity, gender identity outside of cis-normative labels) was a pre-planned omission designed to protect participant anonymity. UAB Medicine has one of the most diverse clinical faculty in the United States, and we enrolled the first consecutive 190 participants who agreed to participate, regardless of race, ethnicity, or multilingualism. As our group previously published, it is important to consider these factors in fitness-for-duty evaluations because they are known to influence test performance (3). Future studies designed to establish national, multi-site representative physician norms will have to address race, ethnicity, primary language/multilingualism, gender identity, and geographical representation (3). Strengths of the study, however, include the largest sample size of healthy physicians to date evaluated with widely used neuropsychological measures.

There are several areas where future research could be helpful before broad public or medical center policies can be adopted. First, increased focus is needed on the relationship between the cognitive performance of at-risk and referred physicians with work-related errors and medical board adjudication outcomes. The development of a multisite, normative database that captures different geographic regions of the United States, different types of medical practice (medical centers, VA, community clinics, private practice, etc.), and a larger number of physicians from the four medical specialty groups need to be recruited. Research on return-to-work protocols is equally important, particularly for various causes of impairment, such as depression, burnout, substance use, or other reversible causes of cognitive decline. Ethical considerations pertaining to the use of physician-based norms pertains to the representative nature of the sample and how well it matches with the referred physician. Our data suggest that physician-based norms, compared to norms from the non-physician population, are superior in physician fitness-for-duty evaluations because of the similar education and occupational history. Even if there are deviations or nuances across physicians, their training will be more consistent than with norms derived from the general public. Our study is therefore a useful model for future research where the normative sample can be expanded to further increase generalizability.

In summary, we provide normative data of healthy physicians, ages 35–65, practicing at a single academic medical center in the United States. On average, physicians perform above the general public across cognitive tests. While age is modestly associated with lower cognitive performance, our cohort of 60-to-65-year-old physicians still outperform the general public. We recommend that medical and surgical specialty groups determine discipline-relevant thresholds for practice based on physician-derived data.

## Data Availability

The raw data supporting the conclusions of this article will be made available by the authors, without undue reservation.
